# Further Validation of a Dutch Translation of the Sussex Oxford Compassion for the Self Scale in Samples of Crisis Line Volunteers, Military Personnel and Nursing Students

**DOI:** 10.3389/fpsyg.2022.895850

**Published:** 2022-07-04

**Authors:** Eva de Krijger, Renate Willems, Peter ten Klooster, Ellen Bakker, Harald Miedema, Constance Drossaert, Ernst Bohlmeijer

**Affiliations:** ^1^Brain Research and Innovation Centre, Utrecht, Netherlands; ^2^Rotterdam University of Applied Science, Research Centre Innovations in Care, Rotterdam, Netherlands; ^3^Department of Psychology, Health and Technology, University of Twente, Enschede, Netherlands

**Keywords:** self-compassion, validation, self-report, SOCS-S, measure

## Abstract

Self-compassion is considered an important, transdiagnostic factor for mental health. The Sussex Oxford Compassion for the Self Scale (SOCS-S) is a recently developed comprehensive measure of self-compassion, that was found to have promising psychometric properties among health care staff and university students in the initial validation study. The aim of this study is the further psychometric evaluation of a Dutch translation of the SOCS-S in different populations and settings. The SOCS-S was administered in three different Dutch samples [crisis line volunteers (*n* = 560), military personnel (*n* = 244) and nursing students (*n* = 255)]. The results confirm the five-factor structure of the SOCS-S and its reliability and criterion and convergent validity across the samples. Measurement invariance was demonstrated for gender in two samples and for age in all three samples, but not across professions. Finally, the SOCS-S was found to explain additional variance in mental health in comparison to a widely used self-compassion measure (SCS-SF).

## Introduction

Mental illness has been estimated to affect about 20% of the adult population each year (Steel et al., 2014), and is one of the leading causes of disability worldwide (Vigo et al., 2016). Furthermore, research supports the notion that individuals free of mental illness do not necessarily experience mental well-being (Iasiello and van Agteren, 2020). Mental wellbeing and mental illness appear to represent two related but distinct continua of mental health, instead of the extreme ends of one single continuum. Research suggests that about one in five adults is free of mental illness but also experiencing suboptimal well-being, i.e., non-flourishing (Keyes, 2005). It has also been found that people with mental illness and people with suboptimal well-being experience similar levels of disability. Both groups report considerably reduced life-satisfaction, more limitations in daily life activities and more loss of (or cutback on) workdays than people with “complete mental health” (Keyes, 2005).

Due to the high prevalence of mental health issues and their personal and societal consequences, there is growing interest in transdiagnostic factors that underlie and maintain mental illness and a lack of flourishing. One relevant transdiagnostic, positive factor is self-compassion (MacBeth and Gumley, 2012; Zessin et al., 2015). Self-compassion can be defined as a supportive and adaptive way of responding to oneself in times of pain or difficulty. Neff (2003a) discerns three components of self-compassion: (1) being kind and understanding instead of harshly critical, (2) being mindfully aware of the pain or difficulty instead of shutting it out, and (3) recognizing the common humanity of pain and difficulty and feeling unified with other human beings because of it instead of experiencing feelings of isolation (Neff, 2003a). Other authors have not offered a separate definition for self-compassion but instead assume that self-compassion is part of the larger construct of compassion, that includes both self- and other-directed compassion. Gilbert (2009), for example, defines compassion as a sensitivity to suffering coupled with the motivation to prevent or relieve it and proposes six key elements: sensitivity, care for wellbeing, sympathy, empathy, non-judgment, and distress tolerance (Gilbert, 2009). Finally, Feldman and Kuyken (2011) define compassion as an orientation of mind that recognizes pain and the universality of pain in human experience and the capacity to meet that pain with kindness, empathy, equanimity and patience (Feldman and Kuyken, 2011).

In the past years many studies found evidence for a negative relationship between self-compassion and distress or mental illness in response to stressful circumstances (Pace et al., 2009; MacBeth and Gumley, 2012; Finlay-Jones et al., 2015; Zeller et al., 2015; Galla, 2016; Barlow et al., 2017; Stutts et al., 2018; Hughes et al., 2021). Other studies demonstrated a relationship between self-compassion and various indicators of mental well-being such as subjective wellbeing, life-satisfaction, job-satisfaction, social connectedness and emotional intelligence (Neff, 2003b; Neff et al., 2007; Abaci and Arda, 2013; Bluth and Blanton, 2014; Zessin et al., 2015; Galla, 2016; McKay and Walker, 2021). The positive relationship between self-compassion and mental health can be explained by various processes such as adaptive emotion regulation (Inwood and Ferrari, 2018), self-reassurance (Sommers-Spijkerman et al., 2018), self-regulation and goal setting (Neff, 2003a; Neff et al., 2005; Williams et al., 2008). There is also a growing body of evidence demonstrating the effects of compassion-based interventions on mental health (Kirby, 2016; Kirby et al., 2017; Sommers-Spijkerman et al., 2021).

However, an important issue in self-compassion research is the variety of conceptualizations of this construct, and the lack of measures that comprehensively capture it (Strauss et al., 2016). Widely used measures of self-compassion are the Self-Compassion Scale (SCS) (Neff, 2003b) and its short form variant (SCS-SF) (Raes et al., 2011), measuring six dimensions of self-compassion (i.e. Self-Kindness, Self-Judgment, Common Humanity, Isolation, Mindfulness, and Over-Identification). However, these instruments have been subject of discussion (Muris and Petrocchi, 2016; Muris et al., 2016; Neff, 2016a,b, 2020; Meng et al., 2019; Muris and Otgaar, 2020). One of the reasons for this discussion is that the six-factor structure of the SCS could not always be confirmed in empirical studies. It has been argued that the SCS and the SCS-SF rather reflect two dimensions: a negative dimension, “self-criticism,” and a positive dimension, “self-compassion” (López et al., 2015; Muris and Petrocchi, 2016; Muris et al., 2016; Babenko and Guo, 2019; Halamová et al., 2021). Some authors have argued that especially the latter, positive dimension, measures “true” self-compassion (Muris and Petrocchi, 2016; Muris et al., 2016). Another measure of self-compassion, the self-compassion subscale of the Relational Compassion Scale (RCS) (Hacker, 2008) only focuses on two very specific aspects of self-compassion, namely emotionally connecting with suffering and acting to help (Strauss et al., 2016).

In an effort to unite different conceptualizations and measurements of self-compassion, Strauss et al. (2016) conducted a review of the various conceptualizations of compassion and based on this review defined compassion, referring to both self-compassion and compassion to others, as a cognitive, affective and behavioral process consisting of (1) recognizing suffering, (2) understanding the universality of suffering in human experience, (3) feeling empathy for the person suffering and connecting with the distress, (4) tolerating uncomfortable feelings aroused in response to the suffering person, and (5) motivation to act to alleviate suffering. Based on factor-analytic examinations Gu et al. (2020) found preliminary support for the five-element definition using a combination of existing and newly generated self-report items. Based on this study, the 20-item Sussex-Oxford Compassion for Others scale (SOCS-O) and the Sussex-Oxford Compassion for the Self scale (SOCS-S) were developed. The SOCS-O and SOCS-S were developed with the purpose of addressing the lack of robust and comprehensive compassion measures. The development consisted of 4 stages; (1) item generation by both experts and non-experts, (2) item reduction based on data from a sample of health care staff, (3) validation of the factor structure and evaluation of psychometric properties in a sample of health care staff and (4) cross-validation and evaluation of psychometric properties in a sample of university students. Data from stages 3 and 4 offered support for the five-factor structure and demonstrated adequate reliability (with a Cronbach’s alpha that ranged from 0.75 to 0.93 for total SOCS-S and subscale scores, and a Cronbach’s alpha that ranged from 0.74 to 0.94 for total SOCS-O and subscale scores) and adequate construct validity in the samples of healthcare staff and university students (Gu et al., 2020). The authors noted, however, that the scales required further testing and cross-validation in other relevant populations (Gu et al., 2020).

The first aim of the current study was to develop and study the psychometric qualities of a Dutch version of the SOCS-S and to thereby also further examine the factor-structure, reliability and validity of the SOCS-S. Replication of the initial findings on the psychometric properties of a newly developed questionnaire in different countries and populations and using different validation questionnaires is warranted and will contribute to the evidence-base for the reliability and construct validity of a questionnaire. In our study the SOCS-S was administered in three different Dutch samples: crisis line volunteers, soldiers and nursing students.

The second aim was to assess measurement invariance of the SOCS-S across the three samples and with regard to gender and age. It is important to assess measurement invariance for a questionnaire, because this indicates whether a questionnaire measures the same construct in the same way across different groups. Measurement invariance is for example assessed by testing whether factor loadings, intercepts and residual variances are equivalent across different groups, for example across different gender groups or across groups with different cultural backgrounds. Measurement invariance is an important statistical property of a questionnaire because it is a prerequisite for comparing questionnaire scores across groups (Putnick and Bornstein, 2016).

The third aim was to explore the extent to which the SOCS-S adds to the SCS-SF in terms of explaining additional variance in mental wellbeing and mental distress. If the SOCS-S explains additional variance in mental wellbeing and mental distress over and above the SCS-SF, this offers support for the incremental validity of the SOCS-S, with respect to the prediction of level of mental wellbeing and mental distress. This would suggest that the SOCS-S provides unique information about people’s level of mental wellbeing and mental distress relative to that which is offered by an existing test of the same construct. Furthermore, if our factor analysis supports the five-factor structure of the SOCS-S, we aim to also examine the five different subscales of the SOCS-S, and whether and to which extent they uniquely contribute to the SCS-SF in terms of explaining additional variance in mental wellbeing and mental distress. We aim to do so because this could provide additional insight into the relative relevance of the five factors with respect to mental wellbeing and distress.

## Methods

### Participants and Procedure

Data for the current study were derived from three research projects, focusing on exploring the relationship of self-compassion and mental health in three different populations: crisis line volunteers, military personnel, and nursing students. These populations were chosen because of their differences in composition and characteristics. Volunteers carry out their task unpaid and on a voluntary basis, for a few hours a week. Military personnel perform their work professionally, sometimes 24/7. Nursing students (first year) are still in training, they are generally younger than the participants in the other populations and have not yet worked in professional practice.

On the other hand, these populations were also chosen because they can be assumed to share an important common characteristic: a strong (social) sense of responsibility and service. After all, different studies underscore the importance of self-compassion for populations that carry a responsibility for the welfare of others, and are focused on taking care of/serving others (Lloyd et al., 2019; Cassidy and McLaughlin, 2021).

Furthermore, crisis-line volunteers and soldiers are regularly confronted with the suffering of others. At this stage of their training, nursing students are generally not yet directly confronted with suffering, but indirectly through reading case histories, talking to qualified nurses, and talking to patients from various sectors (general hospital, nursing homes and psychiatric hospitals). The studies were approved by the ethical committee of the Faculty of Behavioral and Management studies (BMS) of University of Twente (approval number: 191275.

### Sample 1: Crisis Line Volunteers (*n* = 560)

The first sample consisted of volunteers from the “Listen line,” a Dutch crisis line service, run by 1,400 crisis line volunteers. These volunteers are trained to provide a non-judgmental, active listening service to callers; people who cannot or do not want to use professional care (IFOTES, 2020). All crisis line volunteers of the Listen line (*N* = 1405) received a link to the questionnaire by e-mail from their management. The respondents were informed about the aims of the survey and provided active (online) informed consent. After the respondents had given their consent, they continued to fill out the (anonymous) questionnaire. The questionnaire was completed by 560 volunteers (response rate 40%). The mean age of this group was 63 years (*SD* = 11, age range: 22–87 years) and a majority (72%) was female.

### Sample 2: Military Personnel (*n* = 244)

The second sample consisted of military personnel. A sample of 1200 soldiers was randomly selected from the personnel file of the Dutch ministry of Defense. These 1200 soldiers received an email with a link to the questionnaire. The questionnaire was preceded by an information letter and consent form that required active online consent. Only those respondents that provided consent were able to continue to the questionnaire. The questionnaire was completed by 244 soldiers (response rate 20%). The mean age in the total sample was 44 years (*SD* = 10.5, age range: 20–61 years) and 85% were male.

### Sample 3: Nursing Students (*n* = 255)

The third sample consisted of first year nursing students, recruited from the Rotterdam University of Applied Sciences. All students received a link to the questionnaire (*N* = 624). The students were informed about this study by letter. Students could indicate in the questionnaire whether the results could be used for research. The questionnaire was fully completed by 255 students (response rate 41%). The mean age of this group was 19 years (*SD* = 4, age range: 17–50 years) and 92% were female. After completing the questionnaire, students could print out their own scores and discuss them with their study coach.

For all three samples, data were collected using the online survey tool LimeSurvey.

### Measures

Participants answered Dutch versions of all measurements.

### Self-Compassion

Self-compassion was measured with two questionnaires that were administered in each of the three samples.

#### Sussex-Oxford Compassion for the Self Scale

The SOCS-S (Gu et al., 2020) is a 20-item self-report measure, designed to assess self-compassion. The SOCS-S contains five subscales and items are scored on a 5-point Likert scale, ranging from 1 (almost never) to 5 (almost always). Appendix Table 1 provides an overview of these subscales and their items. Gu et al. (2020), together with experts from different countries and continents, formulated items for the questionnaire based on the definition of Strauss et al. (2016). After confirmatory factor analysis (CFA) confirmed the presence of the five theorized elements of compassion, they reduced the number of items, choosing four items with the highest factor loadings per element of self-compassion: (1) Recognizing suffering, (2) Understanding the universality of suffering, (3) Feeling for the person suffering, (4) Tolerating uncomfortable feelings, and (5) Acting or being motivated to act to alleviate suffering. Besides a good fit for a correlated five-factor model, a five-factor hierarchical model also proved to fit the data well, indicating that the five factors in turn load on an overarching self-compassion factor. The psychometric quality of both the SOCS-S total scale, as well as the subscales were supported by the findings of the validation study, in terms of internal consistency (Cronbach’s alpha between 0.75 and 0.93), interpretability, absence of floor and ceiling effects, and convergent and discriminant validity (Gu et al., 2020). Convergent validity was studied by computing correlations with two existing measures of self-compassion, a measure of dispositional empathy, a measure of symptoms of depression, anxiety and stress, a measure of work-related burn-out and a measure of positive mental well-being. Support for convergent validity was provided by the fact that at least three quarters of the results were consistent with predictions that were made and at least two correlations were large (r ≥ 0.50) (Gu et al., 2020).

#### Self-Compassion Scale-Short Form

The SCS-SF (Raes et al., 2011) is a 12-item self-report measure designed to assess self-compassion. The SCS-SF is the shortened version of the original Self Compassion Scale (SCS) that contains 26 items (Raes et al., 2011). Confirmatory factor analysis on the SCS–SF supported a hierarchical model, representing a single higher-order factor of general self-compassion consisting of six first-order factors (self-kindness; self-judgment; common humanity; isolation; mindfulness; over-identification) (Raes et al., 2011). The SCS–SF showed to be a reliable and valid alternative to the long-form SCS, especially when looking at overall self-compassion scores. Items are scored on a seven-point Likert scale, ranging from 1 (almost never) to 7 (almost always). In the current study, a Cronbach’s α coefficient of 0.79 was obtained for the crisis line volunteers, 0.83 for the military personnel, and 0.81 for the nursing students, indicating fair to good internal consistency. In comparison; the original validation study demonstrated a Cronbach’s α coefficient of 0.86 (Raes et al., 2011). In the current study we also computed Cronbach’s α separately for the positive and the negative items of the SCS-SF; for the positive items of the SCS-SF, a Cronbach’s α of 0.73 was found for crisis line volunteers, 0.82 for soldiers, and 0.78 for nursing students. For the negative items of the SCS-SF, a Cronbach’s α of 0.83 was found for the crisis line volunteers, 0.85 for the soldiers, and 0.87 for the nursing students.

### Distress

Distress was measured with a different questionnaire in each sample, because data for the current study was drawn from three separate studies.

#### Subscale Distress of the Four-Dimensional Symptom Questionnaire (4DSQ; Sample 1)

The subscale distress of the 4DSQ (Terluin et al., 2004) contains 16 items that are scored on a five-point Likert scale, ranging from 1 (never) to 5 (always). This scale was completed in the sample of crisis line volunteers. A Cronbach’s α coefficient of 0.89 was obtained for this sample, indicating good internal consistency. In comparison; the original validation study demonstrated a Cronbach’s α coefficient of 0.90 (Terluin et al., 2004).

#### Generalized Anxiety Disorder-7 (GAD-7; Sample 2)

The GAD-7 (Donker et al., 2011) contains seven items that are scored on a 4-point Likert scale, ranging from 0 (never) to 3 (almost every day). This measure was completed in the sample of military personnel. A Cronbach’s α coefficient of 0.85 was obtained for this sample, indicating good internal consistency. In comparison; the original validation study demonstrated a Cronbach’s α coefficient of 0.86 (Donker et al., 2011).

#### Distress Screener (DS; Sample 3)

The Distress Screener (Braam et al., 2009) contains 3 items that are scored on a 3-point Likert scale, ranging from 0 (no) to 2 (regularly/often). This screener was completed in the sample of nursing students. A Cronbach’s α coefficient of 0.78 was obtained for this sample, indicating acceptable internal consistency. In comparison; the original validation study demonstrated a Cronbach’s α coefficient of 0.83 (Braam et al., 2009).

### Mental Wellbeing

#### Mental Health Continuum-Short Form (MHC-SF; Samples 1 and 2)

The MHC-SF (Lamers et al., 2011) is a 14-item self-report measure that is designed to assess mental well-being. Some sample items are: “During the past month, how often did you feel happy?,” and “During the past month, how often did you feel confident to think or express your own ideas and opinions?” Items are rated on a 6-point response scale (never, once or twice a month, about once a week, two or three times a week, almost every day, every day). This measure was completed in both the sample of crisis line volunteers and the sample of military personnel. In the sample of crisis line volunteers, a 5-point Likert scale was mistakenly used, omitting the answer “two or three times a week.” Nevertheless, a good Cronbach’s α coefficient of 0.89 was obtained for the total scale. In addition, in the sample of military personnel, in which the complete Likert scale was provided, the correlation between the MHC-SF and the SCS-SF was 0.40 and for crisis line volunteers the correlation was 0.41. Therefore, we decided to include the results that were obtained in our analyses. For the sample of military personnel, a Cronbach’s alpha coefficient of 0.92 was obtained for the total scale, indicating excellent internal consistency. In comparison; the original validation study demonstrated a Cronbach’s α coefficient of 0.89 (Lamers et al., 2011).

### Engagement

#### Utrecht Student Engagement Scale (UBES-S-9; Sample 3)

The UBES-S-9 (Schaufeli and Bakker, 2003) is a 9-item self-report measure of study engagement. Items are rated on an 7-point Likert scale from 0 (never) to 6 (always). This measure was completed in the sample of nursing students. A Cronbach’s α coefficient of 0.85 was obtained for this sample in the current study, indicating good internal consistency. In comparison; the original validation study demonstrated a Cronbach’s α coefficient of 0.84 (Schaufeli and Bakker, 2003).

### Translation Process of the SOCS-S

To cross-culturally translate the SOCS-S into Dutch, the forward-backward translation method was used (Beaton et al., 2000). In stage 1, three of the authors independently provided a Dutch translation of the SOCS-S. In stage 2 the three authors discussed the three translations from stage 1 to reach consensus on one common translation. In stage 3 a native English speaker, who was blinded to the original English version of the SOCS-S, translated the Dutch translation from stage 2 back into English. In stage 4, the forward and backward translation were compared, and differences were discussed by the four translators and an expert on the concept of self-compassion to reach consensus on the final Dutch translation of the SOCS-S.

### Statistical Analyses

Statistical analyses were conducted using SPSS version 26 and AMOS version 25.

### Psychometric Properties

To examine the underlying *factor structure*, confirmatory factor analysis (CFA) was conducted using the same three models tested by Gu et al. (2020): (a) a single factor model in which all items are direct indicators of self-compassion, to examine whether the items measure a strictly unidimensional construct; (b) a correlated five-factor model, with items loading on their respective factor from the five proposed elements of compassion; and (c) a five-factor hierarchical model, to examine the degree to which the five factors are elements of an overarching construct of self-compassion. Structural equation modeling (SEM) with maximum likelihood estimation was used. A plausible model has low, preferably non-significant, chi-square (χ^2^) values. However, the χ^2^ test is overly sensitive to misfit when the sample size is large (> 200), causing difficulty in obtaining non-significant levels (Kline, 2016). Therefore, four additional fit indices were used to indicate model fit: (1) Root Mean Square Error of Approximation (RMSEA): an absolute fit index scaled as a badness-of-fit statistic where a value of zero indicates the best result, (2) Standardized Root Means Square Residual (SRMR): an absolute fit index that is a badness-of-fit statistic, represents the mean value across all standardized residuals, (3) Comparative Fit Index (CFI): a comparison of the independent model (i.e., observed variables are unrelated) to the estimated model, taking sample size into account, and (4) Akaike Information Criterion (AIC): measures goodness of fit with a penalty on the number of parameters included. This criterion can be used to compare different models, with lower values indicating better fit. These fit indices may each be influenced by numerous factors, such as sample size, data distribution and model complexity and specifications (Schermelleh-Engel et al., 2003). Therefore, we used both liberal and conservative cut-off points for acceptable fit for the CFI, RMSEA, and SRMR: the CFI should be close to or greater than 0.90 (liberal) or 0.95 (conservative), RMSEA should be 0.10 or less (liberal) or 0.06 or less (conservative), and SRMR should be less than 0.10 (liberal) or 0.05 (conservative) (Kline, 2016).

*Internal consistency* was assessed using Cronbach’s alpha (α). Because Cronbach’s α underestimates reliability when items are not tau-equivalent (i.e., do not have equal factor loadings) (Sijtsma, 2009; Deng and Chan, 2017), McDonald’s omega (ω) was also calculated, as it provides a more realistic estimate of the reliability of congeneric measures. Internal consistency was considered adequate when above 0.70 (Cicchetti, 1994).

*Criterion validity* was tested by examining the correlation between the SOCS-S and the SCS-SF. The SCS-SF is one of the most widely used instruments to measure self-compassion. Since the SOCS-S was designed to measure the construct of self-compassion as well, we expected strong correlations (> 0.50) between the SCS-SF and the SOCS-S. Furthermore, in response to recent evidence suggesting a two-factor structure of the SCS-SF, namely self-compassion (positive items) and self-criticism (negative items), we explored the correlations between the SOCS-S and these two factors of the SCS-SF (López et al., 2015; Babenko and Guo, 2019).

*Convergent validity* was tested by examining the pattern of correlations between the SOCS-S and the scales to measure mental wellbeing (MHC-SF, UBES-S-9), and the scales to measure distress (4DSQ, GAD-7 and the DS). Based on evidence for a moderate positive correlation between self-compassion and mental wellbeing and engagement (Zessin et al., 2015), we hypothesized moderate positive correlations between scores on the SOCS-S and scores on scales that measure mental wellbeing (MHC-SF and the UBES-S-9). Based on evidence for a moderate negative correlation between self-compassion and distress (Chio et al., 2021), we hypothesized moderate negative correlations between scores on the SOCS-S and scores on the scales that measured distress (4DSQ, GAD-7 and DS).

### Measurement Invariance

To assess measurement invariance of the best fitting model, three multi-group CFA analyses were performed regarding the three different samples, comparing males and females per sample, and age classes per sample. We determined the mean age for each sample and created two age groups based on the mean split: one group up to the mean age and one group above the mean age. The following four increasingly stringent factor models were tested in the multi-group analyses:

1. Unconstrained model (configural invariance): This baseline model fits the basic model structure across the respective groups simultaneously and has no restrictions on estimated parameters across groups.
2. Model A: Invariant measurement weights (metric invariance). This model tests possible differences on the measurement models. For this, measurement weights (i.e., factor loadings) were fixed to be equal across the groups.
3. Model B: Invariant measurement weights and intercepts (scalar invariance). In this model both measurement weights and measurement intercepts (i.e., mean values) were fixed to be equal across groups.
4. Model C: Invariant measurement weights, intercepts, and structural covariances (measurement and structural invariance). This model tests both possible changes on the measurement and the structural models. In this model measurement weights, measurement intercepts, and structural covariances (i.e., correlations between factors) were fixed to be equal across groups.

In testing these models, we tested whether at each level of invariance a more stringent model still has an acceptable/good fit, and not a significantly worse fit than the previous less stringent model. For the multigroup analysis, the same fit indices and criteria were used as for the CFA analyses. Because χ^2^ difference tests (Δχ^2^) are sensitive to sample size, this test was only used for descriptive purposes, and the absence of relevant changes in CFI values (ΔCFI) > 0.01 along with changes in RMSEA values (ΔRMSEA) > 0.015, or SRMR values (ΔSRMR) > 0.030 between increasingly restrictive models was seen as evidence for sufficient measurement invariance (Cheung and Rensvold, 2002).

### Incremental Validity

Finally, a set of hierarchical blockwise multiple regression analyses were conducted to explore whether the SOCS-S could add significantly to the prediction of mental wellbeing and distress, over and above the SCS-SF.

## Results

### Psychometric Properties

#### Confirmatory Factor Analyses

CFA analyses were conducted to examine the degree to which the three defined factor models fit the data in each sample. The fit indices of the four CFA models are shown in Table 1.

**TABLE 1:** Fit indices for the self-compassion models tested in three samples.

Sample	Model	X^2^, (df)	RMSEA*^a^* (90% CI)	SRMR^b^	CFI^c^	AIC^d^
Crisis line volunteers (*N* = 580)	One-factor	2087.2 (170)	0.14 (0.14–0.15)	0.11	0.73	2167.18
	Five-factor	603.5 (160)	0.07 (0.07–0.08)	0.05	0.94	703.55
	Five factor-hierarchical	708.4 (165)	0.08 (0.07–0.08)	0.07	0.92	798.38
Military personnel (*N* = 244)	One-factor	747.9 (170)	0.12 (0.11–0.13)	0.08	0.81	827.95
	Five-factor	393.0 (160)	0.08 (0.07–0.09)	0.05	0.92	492.98
	Five factor-hierarchical	435.7 (165)	0.08 (0.07–0.09)	0.07	0.91	525.70
Nursing students (*N* = 255)	One-factor	852.4 (170)	0.13 (0.11–0.13)	0.11	0.73	932.40
	Five-factor	357.8 (160)	0.07 (0.06–0.08)	0.06	0.92	457.84
	Five factor-hierarchical	378.0 (165)	0.07 (0.06–0.08)	0.07	0.92	469.24

*^a^RMSEA, Root Mean Square Error of Approximation. ^b^Standardized Root Means Square Residual. ^c^Comparative Fit Index. ^d^Akaike Information Criterion.*

**FIGURE 1:**
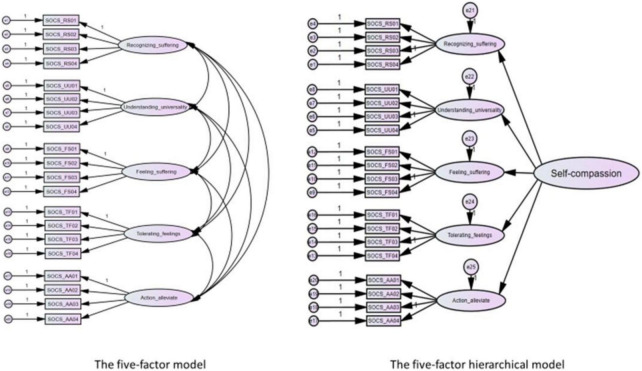
The five factor model and the five-factor hierarchical model of the SOCS-S.

In all three samples, both the correlated five-factor model and the five-factor hierarchical model (Figure 1) showed acceptable fit according to the liberal fit indices, with the five-factor model showing a slightly better fit than the five-factor hierarchical model. As expected, the one-factor model demonstrated a poor fit in all three samples, suggesting that the items of the SOCS-S are not direct indicators of a unidimensional self-compassion factor. In both the five-factor model (range 0.48–0.90) and the five-factor hierarchical model (range 0.47–0.90), all standardized factor loadings were strong and significant (Appendix Table 1). Pearson correlations among the subscales were moderate to strong and significant across the different samples (Table 2). The factor loadings were very similar in both the correlated five-factor and hierarchical five-factor models (Appendix Table 1). While the correlated five factor model and the five factor hierarchical model both show acceptable fit and factor loadings are very similar for these two models, the fit indices point to the correlated five factor model as the model that fits our data best. Based on these results, we have retained this model for the further psychometric analyses that we performed.

**TABLE 2:** Pearson’s correlations between self-compassion subscales in three samples.

		UU^b^	FS^c^	TF^d^	AA^e^
SOCS-S RS^a^	Crisis line volunteers (*N* = 560)	0.47***	0.53***	0.55***	0.48***
	Military personnel (*N* = 244)	0.53***	0.65***	0.67***	0.60***
	Nursing students (*N* = 255)	0.35***	0.33***	0.32***	0.30***
SOCS-S UU	Crisis line volunteers (*N* = 560)		0.38***	0.43***	0.35***
	Military personnel (*N* = 244)		0.46***	0.50***	0.44***
	Nursing students (*N* = 255)		0.30***	0.32***	0.29***
SOCS-S FS	Crisis line volunteers (*N* = 560)			0.80***	0.84***
	Military personnel (*N* = 244.)			0.84***	0.89***
	Nursing students (*N* = 255)			0.78***	0.87***
SOCS-S TF	Crisis line volunteers (*N* = 560)				0.74***
	Military personnel (*N* = 244)				0.79***
	Nursing students (*N* = 255)				0.73***

^a^RS, recognizing suffering. ^b^UU, understanding universality. ^c^FS, feeling one’s own suffering. ^d^TF, tolerating feelings. ^e^AA, action to alleviate suffering. ***p < 0.001.

#### Internal Consistency

Omega total estimates ranged from 0.68 to 0.88 for the SOCS-S subscales across the samples (Table 3). Cronbach’s alpha values were similar to the omega values. Observed values were all adequate for measures of psychological constructs, with the exception of the reliability estimates (for the recognizing suffering subscale (α = 0.67, ω = 0.68) in nursing students.

**TABLE 3:** Cronbach’s alpha and McDonald’s omega total coefficients for SOCS-S subscales in all validation samples.

	Crisis line volunteers (*N* = 560)		Military personnel (*N* = 244)		Nursing students (*N* = 255)	
	Alpha	Omega	Alpha	Omega	Alpha	Omega
SOCS-S RS^a^	0.82	0.82	0.81	0.82	0.67	0.68
SOCS-S UU^b^	0.86	0.86	0.80	0.80	0.83	0.83
SOCS-S FS^c^	0.85	0.85	0.85	0.85	0.83	0.84
SOCS-S TF^d^	0.85	0.85	0.82	0.83	0.76	0.76
SOCS-S AA^e^	0.87	0.88	0.88	0.88	0.83	0.85

*^a^RS, recognizing suffering. ^b^UU, understanding universality. ^c^FS, feeling one’s own suffering. ^d^TF, tolerating feelings. ^e^AA, action to alleviate.*

#### Criterion and Convergent Validity

Table 4 shows the correlations between the results on the five subscales of the SOCS-S and an existing and often used measure of self-compassion (SCS-SF), different measures of mental wellbeing and different measures of distress.

**TABLE 4:** Correlations coefficients between scores on the subscales of the SOCS-S with the SCS-SF (and subscales), mental wellbeing (and subscales) and distress.

				Self-compassion			Mental wellbeing		Distress		
		Age	Gender^f^	SCS-SF total	SCS-SF Positive	SCS-SF Negative	MHC-SF Positive mental health	UBES-S Engagement students	4DCL	GAD-7	Distress screener
SOCS-S RS^a^	Crisis line volunteers (*N* = 543)	0.02	0.12**	0.27**	0.32**	−0.13**	0.17**		−0.10*		
	Military personnel (*N* = 244)	0.12	0.02	0.55**	0.51**	−0.36**	0.24**			−0.20**	
	Nursing students (*N* = 255)	0.01	0.10	0.18**	0.18**	−0.12*		0.18**			0.07
SOCS-S UU^b^	Crisis line volunteers (*N* = 543)	0.00	0.05	0.29**	0.38**	−0.12*	0.21**		−0.03		
	Military personnel (*N* = 244)	0.12	−0.01	0.48**	0.49**	−0.28**	0.25**			−0.25**	
	Nursing students (*N* = 255)	0.12	−0.05	0.37**	0.43**	−0.15*		0.14*			−0.10
SOCS-S FS^c^	Crisis line volunteers (*N* = 543)	0.07	0.05	0.62**	0.49**	−0.50**	0.41**		−0.31**		
	Military personnel (*N* = 244)	0.11	−0.02	0.70**	0.64**	−0.52**	0.34**			−0.37**	
	Nursing students (*N* = 255)	0.09	−0.09	0.63**	0.65**	−0.42**		0.21**			−0.43**
SOCS-S TF^d^	Crisis line volunteers (*N* = 543)	0.10*	−0.05	0.60**	0.49**	−0.47**	0.40**		−0.33**		
	Military personnel (*N* = 244)	0.20**	0.06	0.73**	0.71**	−0.52**	0.32**			−0.38**	
	Nursing students (*N* = 255)	0.08	−0.07	0.59**	0.62**	−0.38**		0.24**			−0.39**
SOCS-S UU^e^	Crisis line volunteers (*N* = 543)	0.05	0.07	0.57**	0.46**	−0.45**	0.41**		−0.32**		
	Military personnel (*N* = 244)	0.09	−0.06	0.68**	0.61**	−0.52**	0.34**			−0.38**	
	Nursing students (*N* = 255)	0.07	−0.07	0.58**	0.63**	−0.36**		0.28**			−0.44**

^a^RS, recognizing suffering. ^b^UU, understanding universality. ^c^FS, feeling suffering. ^d^TF, tolerating feelings. ^e^AA, action to alleviate suffering. ^f^gender: 1, man, 2, woman, 3, other. **p < 0.01, *p < 0.05.

In line with hypotheses, the subscales “feeling suffering,” “tolerating feelings” and “action to alleviate suffering” demonstrated significant and strong correlations with the SCS-SF total scale in all three samples (*r* = 0.57 to *r* = 0.73). The same was true for the correlation between the subscale “recognizing suffering” and the SCS-SF total scale in the sample of military personnel, which was also demonstrated to be significant and strong (*r* = 0.55). However, in contrast to hypotheses, the other correlations between the subscales “recognizing suffering” and “understanding universality” were demonstrated to be significant but only weak to moderate in magnitude (*r* = 0.18 to *r* = 0.48).

Furthermore, correlations between the SOCS-S subscales and the “self-compassion” factor of the SCS-SF (SCS-SF positive) tended to be stronger for most subscales (*r* = 0.18 to r = 0.71 in the three different samples) than correlations with the “self-criticism” factor of the SCS-SF (SCS-SF negative) (*r* = 0.12 to *r* = 0.52 in the three different samples).

Furthermore, in line with hypotheses, the subscales “feeling suffering,” “tolerating feelings” and “action to alleviate suffering” demonstrated significant and moderate positive correlations with scores on the MHC-SF (r = 0.32 to r = 0.41). However, contrary to hypotheses, significant but only weak positive correlations were demonstrated between the scores on the subscales “recognizing suffering” and “understanding universality” and scores on the MHC-SF (r = 0.17 to r = 0.25). The correlations between SOCS-S subscale scores and scores on the UBES-S-9 were found to be significant but to range from 0.14 to 0.28 and thereby classify as weak positive correlations (*r* < 0.30), instead of the moderate correlations that were predicted. Overall, the subscales “recognizing suffering” and “understanding universality” of the SOCS-S demonstrated the lowest correlations with the MHC-SF and the UBES-S-9.

Finally, in line with hypotheses, the subscales “feeling suffering,” “tolerating suffering” and “action to alleviate suffering” demonstrated significant and moderate negative correlations with a measure of distress in all three samples (r = –0.31 to r = –0.44). However, contrary to hypotheses, the subscales “recognizing suffering” and “understanding universality” demonstrated weak and some significant but also some non-significant negative correlations with a measure of distress in all three samples (r = –0.03 to r = –0.25).

### Measurement Invariance

Multi-group analyses were conducted to examine the measurement invariance between the three different samples, between men and women, and between different age groups for the correlated five-factor model, as this model showed the best fit across the samples. Table 5 shows the results of these multi-group analyses.

**TABLE 5:** Three multi-group analyses of the five-factor model in: the different samples, gender, and age.

Results of the multi-group analysis: different samples												
	
	Model	X^2^	df	X^2^/df	ΔX^2^	Δdf	CFI	ΔCFI	RMSEA (IC 90%)	ΔRMSEA	SRMR	ΔSRMR
Total sample	Unconstrained	1,354.37	480	2.82			0.93		0.042 (0.039–0.044)		0.05	
	Model A	1,397.29	510	2.74	42.93	30	0.93	0.001	0.041 (0.038–0.043)	−0.001	0.05	0.001
	Model B	1,765.89	550	3.21	368.59***	40	0.90	0.026	0.046 (0.043–0.048)	0.005	0.05	0.000
	Model C	1,875.89	580	3.23	110.00***	30	0.90	0.006	0.046 (0.044–0.048)	0.000	0.07	0.016
**Results of the multi-group analysis: man and woman**												
Crisis line volunteers	Unconstrained	826.24	320	2.58			0.93		0.053 (0.049–0.058)		0.06	
	Model A	842.17	335	2.51	15.93	15	0.93	0.000	0.052 (0.048–0.057)	0.001	0.06	0.001
	Model B	878.80	355	2.48	36.63*	20	0.93	0.002	0.052 (0.047–0.056)	0.000	0.06	0.000
	Model C	914.98	370	2.47	36.17**	15	0.93	0.003	0.051 (0.047–0.056)	0.001	0.06	0.005
Soldiers	Unconstrained	654.17	320	2.04			0.89		0.066 (0.059–0.073)		0.05	
	Model A	664.30	335	1.98	10.12	15	0.90	0.002	0.064 (057–0.071)	0.002	0.05	0.002
	Model B	677.87	355	1.91	13.57	20	0.90	0.002	0.062 (0.055–0.069)	0.000	0.05	0.000
	Model C	706.47	370	1.91	26.60*	15	0.89	0.004	0.062 (0.055–0.069)	0.000	0.06	0.009
Nursing students	Unconstrained^a^	756.19	320	2.36			0.84		0.073 (0.067–0.080)		No fit	
**Results of the multi-group analysis: age group**												
Crisis line volunteers	Unconstrained	840.44	320	2.63			0.93		0.054 (0.050–0.059)		0.06	
	Model A	865.62	335	2.58	25.18*	15	0.93	0.002	0.053 (0.049–0.058)	0.001	0.06	0.002
	Model B	906.14	355	2.55	40.52**	20	0.93	0.003	0.053 (0.049–0.057)	0.001	0.06	0.003
	Model C	937.22	370	2.53	31.09**	15	0.92	0.002	0.053 (0.048–0.057)	0.000	0.07	0.010
Soldiers	Unconstrained	611.61	320	1.91			0.90		0.062 (0.054–0.069)		0.06	
	Model A	617.97	335	1.84	6.36	15	0.91	0.003	0.060 (0.052–0.067)	0.002	0.06	0.001
	Model B	637.71	355	1.80	19.74	20	0.91	0.000	0.058 (0.051–0.065)	0.002	0.06	0.000
	Model C	655.28	370	1.77	17.57	15	0.90	0.001	0.057 (0.050–0.064)	0.001	0.07	0.008
Nursing students	Unconstrained	610.50	320	1.91			0.89		0.060 (0.053–0.067)		0.07	
	Model A	626.34	335	1.87	15.84	15	0.89	0.000	0.059 (0.052–0.066)	0.001	0.07	0.000
	Model B	644.17	355	1.84	27.83	20	0.89	0.003	0.058 (0.051–0.065)	0.001	0.07	0.000
	Model C	679.96	370	1.84	25.79*	15	0.88	0.004	0.058 (0.051–0.064)	0.000	0.07	0.001

^a^Because the unstrained five factor model has no fit in nursing students, the measurement invariance cannot be calculated. Model A, measurement weights. Model B, measurement intercepts. Model C, structural covariances. ***p < 0.001, **p < 0.01, *p < 0.05.

The multigroup analysis to examine measurement invariance between the three different samples revealed that the Δχ^2^ test of model A was not significant and that the Δχ^2^ of models B and C were significant, indicating that invariance is supported on measurement weights but not supported on measurement intercepts and structural covariances. The ΔCFI was also only smaller than 0.01 on model A, indicating that only metric invariance was supported.

For men and women of the crisis line service the Δχ ^2^ test of model A was not significant and the Δχ^2^ test of models B and C were significant. Among the soldiers, the Δχ ^2^ test of models A and B was not significant, while for model C it was. However, because in both crisis line volunteers and soldiers the ΔCFI, the ΔRMSEA, and the ΔSRMR were all below the defined cut-offs for relevant changes, the SOCS-S demonstrated measurement invariance at all levels. Because the group of men in the nursing student sample was too small, we could not determine whether there was measurement variance.

In all three samples, measurement invariance was demonstrated across age groups, because for all models in all samples the ΔCFI < 0.01, the ΔRMSEA < 0.015, and the ΔSRMR < 0.03.

### Incremental Validity

To examine whether the SOCS-S adds to the SCS-SF in terms of explained variance of mental wellbeing (crisis line volunteers and soldiers) and engagement (nursing students), hierarchical blockwise multiple regression analyses were conducted in which the SCS-SF was entered in the first step, and the subscales of the SOCS-S were entered in the second step (Table 6). Table 6 only demonstrates the statistically significant results and omits the results that were not statistically significant.

**TABLE 6:** Summary of multiple regression analysis of the added value of the SOCS-S (subscales) on positive mental health.

	Model		β	*R* ^2^	*R^2^_change_*	*F* (*df1, df2*)
Crisis line volunteers	1	SCS-SF^a^	0.41***	0.17		(1, 558) = 112.5***
	2	SCS-SF^a^	0.20***	0.23	0.06	(5, 553) = 27.2)***
		SOCS-S RS^b,c^	−0.10*			
		SOCS-S UU^d^	0.06			
		SOCS-S FS^e^	0.07			
		SOCS-S TF^f^	0.13			
		SOCS-S AA^g^	0.17*			
Military personnel	1	SCS-SF^a^	0.36***	0.13		(1, 242) = 35.81***
	2	SCS-SF^a^	0.21*	0.15	0.02	(5, 237) = 7.00***
		SOCS-S RS^b,c^	−0.04			
		SOCS-S UU^d^	0.08			
		SOCS-S FS^e^	0.07			
		SOCS-S TF^f^	0.01			
		SOCS-S AA^g^	0.11			

^a^SCS-SF, Self-Compassion Scale Short Form. ^b^SOCS-S, the Sussex Oxford Compassion for the Self Scale. ^c^SOCS-RS, subscale SOCS-S recognizing suffering. ^d^SOCS-UU, subscale SOCS-S understanding universality of suffering. ^e^SOCS-FS, subscale SOCS-S feeling one’s own suffering. ^f^SOCS-TF, subscale SOCS-S tolerating uncomfortable feelings. ^g^SOCS-AA, subscale SOCS-S acting or being motivated to act to alleviate suffering. ***p < 0.001. *p < 0.05.

The results revealed that the SOCS-S added significantly to the SCS-SF in terms of explained variance of positive mental health in the samples of crisis line volunteers and military personnel. Among crisis line volunteers, the explained variance of mental well-being increased by 6% from 17 to 23%. Among military personnel, the explained variance of mental well-being increased by 2%, from 13 to 15%. Among crisis line volunteers, the subscales “recognizing suffering” and “acting or being motivated to act to alleviate suffering” in particular appeared to have added value as independent explaining variables. For military personnel, the subscales of the SOCS-S appeared to have added value in total, but none of the subscales independently contributed significantly to the explained variance of mental wellbeing. In the sample of nursing students, the SOCS-S demonstrated no added value with regard to explained variance of engagement.

To examine whether the SOCS-S added to the SCS-SF in terms of explained variance of distress, three additional hierarchical multiple regression analyses were applied, again with de SOCS-S subscales added in step 2. The results revealed that the SOCS-S did not add to the SCS-SF with regard to explained variance of distress in any of the three samples.

## Discussion

The current study focused on the psychometric assessment of the Sussex-Oxford Compassion for the Self Scale (SOCS-S) (Gu et al., 2020), a recently developed, comprehensive measure of self-compassion. In the current study the SOCS-S was administered in three different samples: crisis line volunteers, military personnel and nursing students. The aims of the current study were (1) to assess the factor structure, reliability and construct validity of the SOCS-S in each sample, (2) to assess measurement invariance of the SOCS-S S across different groups, and (3) to explore the extent to which the SOCS-S adds to the SCS-SF in terms of explaining mental wellbeing and distress.

First, with regard to the factorial validity of the SOCS-S, confirmatory factor analyses showed support for the proposed five factor structure of the SOCS-S. The correlated five factor model fitted best with the data from our three samples. Pearson correlations between subscales were found to be moderate to strong. These results suggest that the SOCS-S consists of five correlated subscales each contributing uniquely to self-compassion. Furthermore, the subscales “recognizing suffering” and “understanding universality” were found to demonstrate the lowest correlations with the other subscales. With regard to internal consistency of the SOCS-S, adequate to good internal consistency was found for all of the subscales of the SOCS-S. One exception concerns the internal consistency of the subscale “recognizing suffering.” This subscale demonstrated unacceptable internal consistency in our sample of nursing students. In the other two samples good internal consistency was demonstrated for this subscale. One explanation might be the fact that our sample of nursing students was considerably younger than the other two samples, and that the extent to which they have been confronted with suffering might be less than for the other two samples. Lastly, we found moderate to strong correlations in the expected directions between the subscales “feeling suffering,” “tolerating suffering” and “action to alleviate suffering” on the one hand and the SCS-SF and measures of mental wellbeing, distress and engagement on the other hand. These findings were in line with our hypotheses and underscored the criterion and convergent validity of these three subscales of the SOCS-S. However, contrary to our predictions the subscales “recognizing suffering” and “understanding universality” were found to correlate in the expected directions but weakly to moderately with the SCS-SF and weakly with measures of mental wellbeing, distress and engagement. These results suggest that these two subscales may contribute to self-compassion in a markedly different manner. This difference appears to make sense because while one cannot have one without the other (e.g., one cannot take care of oneself in the face of suffering or take action to relieve suffering, without recognizing the suffering first), it seems plausible that independently the extent to which one is able to care for oneself in the face of suffering, to tolerate uncomfortable feelings and to take action to relieve the suffering are more strongly related to one’s level of mental wellbeing and distress than the extent to which one is able to recognize suffering and to recognize that “suffering is all around” (universality of suffering). Overall, our findings are in line with the results from the initial validation study (Gu et al., 2020). Therefore, these results offer support for the psychometric properties of the SOCS-S across different samples, thereby adding to the robustness of evidence for the SOCS-S as a psychometrically sound measure for self-compassion.

Secondly, we assessed measurement invariance of the SOCS-S across the three different samples, across gender and across age. Our results showed support for full measurement invariance of the SOCS-S across gender and age, except for the fact that measurement invariance could not be determined across gender for our sample of nursing students because the group of men in this sample was too small. On the other hand, our results showed only partial support for measurement invariance of the SOCS-S across our three samples of interest. This finding is important because measurement invariance assesses whether the construct that is measured has the same structure and meaning in different groups and is therefore a prerequisite for meaningful comparisons between groups (Putnick and Bornstein, 2016). Our results suggest that the construct that is measured by the SOCS-S has the same structure and meaning for men and women and age groups, and that group means of SOCS-S scores can be validly compared across genders and age groups. However, the meaning and structure of this construct seem somewhat different for our three different samples. One explanation might be that the items of the SOCS-S might have a different meaning for military personnel because of the training that they receive which contains an explicit focus on training hardiness. Military training might influence the way military personnel interpret words like “caring.” In this regard it is also important to mention that our sample sizes were severely unbalanced, with the sample of crisis line volunteers being much larger than the other two samples. This is important because violations of invariance might be masked in case of severely unbalanced group size conditions (Yoon and Lai, 2018). We can therefore not exclude the possibility that the partial support for measurement invariance that our results did show, are in fact results of the bias caused by the unbalanced sample sizes. Based on these results, direct comparisons between observed scores in these different samples should be made with care, because these differences could also be dependent on group membership.

Thirdly, we explored the extent to which scores on the SOCS-S add to the SCS-SF in terms of explaining variance in mental wellbeing and distress. Our results showed a modest but significant increase in explained variance of mental wellbeing in two of the samples (crisis line volunteers and military personnel). In the sample of nursing students an increase in explained variance of engagement was not demonstrated. The results in the samples of crisis line volunteers and military personnel suggest that scores on the SOCS-S add to the SCS-SF, in accurately predicting level of mental wellbeing. In the sample of military personnel, it was only the SOCS-S total that added significantly to the SCS-SF in terms of explained variance in mental wellbeing, but in the sample of crisis line volunteers, it was in addition found that specifically the subscales “recognizing suffering” and “acting to alleviate suffering” independently added to the explained variance of mental wellbeing. The subscale “recognizing suffering” appears to differ from the SCS-SF in the sense that the subscale “recognizing suffering” focuses solely on awareness of the suffering (e.g., “I notice when I’m feeling sad or stressed”), while items of the SCS-SF focus on *mindful* awareness of the suffering (e.g., “when something painful happens I try to take a balanced view of the situation”). Our findings suggest that ‘recognizing suffering’ in itself is relevant for mental wellbeing, apart from a mindful reaction to the painful situation. Furthermore, the association between “recognizing suffering” and “mental wellbeing” was negative in the multivariate model, suggesting that “recognizing suffering” in itself, apart from a mindful reaction to the painful situation, actually relates to a *lower* level of mental wellbeing when adjusting for the other elements of self-compassion. This is consistent with what has been shown in neuropsychological research (Klimecki et al., 2013): merely being exposed to suffering (recognizing suffering) is associated with distress and activates brain networks associated with pain. However, self-compassion includes both recognizing suffering and the motivation and action to alleviate it. In this combination, self-compassion is associated with positive feelings such as comfort and affiliation, thus activating brain networks related to reward and affiliation (Klimecki et al., 2013).

The other subscale that uniquely added to the SCS-SF in terms of explained variance of mental wellbeing among crisis line volunteers was “acting to alleviate.” “Acting to alleviate” is not specifically measured within the SCS-SF. The SCS-SF does measure the factor “kindness,” which entails care and understanding, but a factor that specifically focuses on acting to make oneself feel better, is not included in the SCS-SF. Our findings therefore suggests that “acting to alleviate” is a factor that is relevant for mental wellbeing, apart from kindness, care and understanding. This corresponds with several conceptualizations of self-compassion that entail “acting to alleviate” as an important part of self-compassion (Lazarus and Lazarus, 1991; Lama and Thupten, 1995; Kanov et al., 2004; Gilbert, 2009; Goetz et al., 2010). In fact, the conceptualization proposed by Gilbert (2009) stresses the importance of acting to alleviate, by stating that compassion consists of recognizing suffering *coupled with* the motivation to prevent or alleviate suffering and that one of these factors on its own does not equal compassion.

We did not find an increase in explained variance of distress after adding SOCS-S subscale scores to SCS-SF scores in any of the three samples. One explanation might be the fact that the SOCS-S consists of positive items only, because a meta-analysis performed by Muris and Petrocchi (2016) demonstrated that the negative items of the SCS-SF demonstrate larger correlations with mental health problems than the positive items. If positive items correlate lower with mental health problems, than a measure of self-compassion that consists of positive items only, such as the SOCS-S, might shed a different light on the relationship between self-compassion and psychopathology (MacBeth and Gumley, 2012).

### Limitations

The three samples that were included in the current study can be assumed to share an important common characteristic; a strong (social) sense of responsibility and service. This is one of the reasons why these populations were chosen, but it might limit the extent to which the results of the current study can be generalized to other (community) populations.

Furthermore, as would be expected, the three samples that were included in the current study showed systematic differences in age and gender; the sample of nursing students was considerably younger than the other two samples and consisted of mainly females, whereas the sample of military personnel consisted of mainly males. The fact that measurement invariance was not supported across the three different samples could therefore be caused by the fact that measurement invariance was not supported for different age groups, or the other way around. Systematic differences in age between the three different samples limit a definitive conclusion with regard to this matter.

In addition, response rates were about 40% for the samples of crisis line volunteers and nursing students and about 20% for military personnel. Combined the total response rate was about 32%. Especially for the sample of military personnel there was a considerable amount of non-response, increasing the likelihood that the current results are influenced by self-selection bias (Bethlehem, 2010).

Furthermore, the validation questionnaires that were administered were different across our three samples. Distress was measured with a different questionnaire in each of the three samples, and “mental wellbeing” was measured in two of our samples, but in the third sample we measured “engagement” instead. This inequality in measurement did not form a limitation with regard to assessing convergent validity, but it precludes the possibility of comparing our three samples with regard to level of mental wellbeing and distress.

In addition, the scale that was used to measure mental wellbeing (the MHC-SF) contained an error in the sample of crisis line volunteers. The error consisted of using a 5-point response scale, instead of a 6-point response scale, in which the answer “two or three times a week” was mistakenly left out. Based on the fact that the scale demonstrated good internal consistency, we decided to include these data in our analyses.

Finally, since we performed our CFA analyses with AMOS, we were limited to using normal theory maximum likelihood estimation. Future studies are encouraged to use SEM software that allows the use of more robust estimation methods that are better suited for Likert-type items.

### Conclusions and Future Research

The current study underscores the 5-factor structure, the validity and reliability of the SOCS-S. The results of the current study in addition suggest that the subscales “recognizing suffering” and “understanding universality” are less strongly correlated with mental wellbeing and mental distress than the other three subscales. Future research focusing on the independent subscales of the SOCS-S is warranted to see if these findings will be replicated. In addition, the study demonstrates full measurement invariance of the SOCS-S across gender. Our findings also suggest that the SOCS-S explains some additional variance of mental wellbeing in comparison with a widely used instrument for self-compassion, the SCS-SF, in two of our samples. Measurement invariance was not supported for the SOCS-S across age and across our three different samples. Direct comparisons between observed scores of different age groups and different professions should therefore be made with care. Future research focusing on different age groups and/or samples of different professions is warranted to improve understanding of these differences. Furthermore, while in the current study measurement invariance for age was studied by comparing different age groups, recent studies demonstrate the value of estimating measurement invariance for age using moderated factor analysis during which the age variable is considered as a moderating variable that interacts with the latent variable (Molenaar, 2020; Jovanović et al., 2022). Future research employing this method of estimating measurement invariance is recommended to improve understanding of the differences that were shown in the current study. Finally, future studies are recommended to make use of software that enables confirmatory factor analysis with more robust estimation methods.

## Data Availability Statement

The raw data supporting the conclusions of this article will be made available by the authors, without undue reservation.

## Author Contributions

EK, RW, PK, HM, CD, and ErB conceptualized the manuscript and drafted the manuscript. EK, RW, and PK conducted all the statistical analyses. EK, RW, ElB, and HM collected the data. All authors were involved in the design of the study and final approval for the published article and agreed to be accountable for all aspects of the work in ensuring that questions related to the accuracy or integrity of any part of the work are appropriately investigated.

## Conflict of Interest

The authors declare that the research was conducted in the absence of any commercial or financial relationships that could be construed as a potential conflict of interest.

## Publisher’s Note

All claims expressed in this article are solely those of the authors and do not necessarily represent those of their affiliated organizations, or those of the publisher, the editors and the reviewers. Any product that may be evaluated in this article, or claim that may be made by its manufacturer, is not guaranteed or endorsed by the publisher.

## Appendix

**TABLE A1:** Factor loadings of all items on the five-factor model.

			Five-factor model		
			Crisis line volun-teers (*N* = 560)	Veterans (*N* = 244)	Nursing students (*N* = 255)
RS^a^	1.	I’m good at recognising when I’m feeling distressed.	0.71*	0.68*	0.56*
	6.	I notice when I’m feeling distressed.	0.79*	0.73*	0.55*
	11.	I’m quick to notice early signs of distress in myself.	0.75*	0.78*	0.81*
	16.	I recognize signs of suffering in myself.	0.69*	0.72*	0.48*
UU^b^	2.	I understand that everyone experiences suffering at some point in their lives.	0.68*	0.67*	0.64*
	7.	I understand that feeling upset at times is part of human nature.	0.80*	0.70*	0.74*
	12.	Like me, I know that other people also experience struggles in life.	0.82*	0.74*	0.79*
	17.	I know that we can all feel distressed when things don’t go well in our lives.	0.82*	0.75*	0.81*
FS^c^	3.	When I’m going through a difficult time. I feel kindly toward myself.	0.78*	0.69*	0.80*
	8.	When bad things happen to me, I feel caring toward myself.	0.84*	0.84*	0.86*
	13.	When I’m upset, I try to tune in to how I’m feeling.	0.68*	0.74*	0.55*
	18.	Even when I’m disappointed with myself. I can feel warmly toward myself when I’m in distress.	0.79*	0.80*	0.79*
TF^d^	4.	When I’m upset I try to stay open to my feelings rather than avoid them.	0.74*	0.76*	0.62*
	9.	I connect with my own distress without letting it overwhelm me.	0.79*	0.75*	0.64*
	14.	I connect with my own suffering without judging myself.	0.80*	0.77*	0.73*
	19.	When I’m upset, I can let the emotions be there without feeling overwhelmed.	0.72*	0.64*	0.69*
AA^e^	5.	I try to make myself feel better when I’m distressed, even if I can’t do anything about the cause.	0.59*	0.68*	0.52*
	10.	When I’m going through a difficult time, I try to look after myself.	0.88*	0.90*	0.85*
	15.	When I’m upset, I try to do what’s best for myself.	0.81*	0.78*	0.76*
	20.	When I’m upset, I do my best to take care of myself.	0.90*	0.86*	0.89*

*aRS, recognizing suffering. bUU, understanding universality. cFS, feeling one’s own suffering. dTF, tolerating feelings. eAA, action to alleviate suffering. *p < 0.001.*
